# Assessment of Prenatal Transportation Stress and Sex on Gene Expression Within the Amygdala of Brahman Calves

**DOI:** 10.3390/biology13110915

**Published:** 2024-11-11

**Authors:** Emilie C. Baker, David G. Riley, Rodolfo C. Cardoso, Thomas B. Hairgrove, Charles R. Long, Ronald D. Randel, Thomas H. Welsh

**Affiliations:** 1 Department of Agricultural Sciences, West Texas A&M University, Canyon, TX 79016, USA; ebaker@wtamu.edu; 2 Department of Animal Science, Texas A&M University, College Station, TX 77843, USA; david.riley@ag.tamu.edu (D.G.R.); r.cardoso@tamu.edu (R.C.C.); thomas.hairgrove@ag.tamu.edu (T.B.H.); charles.long@ag.tamu.edu (C.R.L.); r-randel@tamu.edu (R.D.R.); 3 Texas A&M AgriLife Research, College Station, TX 77843, USA; 4 Texas A&M AgriLife Extension, College Station, TX 77843, USA; 5 Texas A&M AgriLife Research, Overton, TX 75684, USA

**Keywords:** amygdala, bovine, gene expression, outliers, prenatal stress

## Abstract

As the amygdala is associated with fear and anxiety, it is important to determine the potential effects of gestational stressors on behavior and stress responses in offspring. The objective of this study was to investigate the effects of prenatal transportation stress on amygdala gene expression in 25-day-old Brahman calves, focusing on sex-specific differences. Amygdala tissue samples from prenatally stressed (PNS) and control bull and heifer calves were analyzed using RNA sequencing. A thorough outlier detection process, utilizing visual inspection of multidimensional scaling plots, robust principal component analysis, and PCAGrid methods, led to the exclusion of 5 of 32 samples from subsequent analyses. Differential expression analysis revealed no significant treatment differences between the control and PNS groups within either sex. However, sex-specific differences in gene expression were identified in both the control and PNS groups. The control group showed seven differentially expressed genes between sexes, while ten were identified between PNS males and females, with seven located on the X chromosome. Among these was the ubiquitin-specific peptidase 9 X-linked gene, which plays a role in neurodevelopmental pathways. When comparing males to females, regardless of treatment, a total of 58 genes were differentially expressed, with 45 showing increased expression in females. Gene enrichment analysis indicated that many differentially expressed genes are associated with infectious disease-related pathways. Future research should explore amygdala size and functional responses to various postnatal stimuli.

## Simple Summary

This study investigated the effects of prenatal transportation stress on gene expression in the amygdala of 25-day-old Brahman calves. Amygdala samples from both prenatally stressed (PNS) and control calves were analyzed. A thorough outlier detection process led to the exclusion of 5 of 32 samples. Overall, gene expression was not significantly altered by prenatal stress. However, notable differences were found between sexes, with seven differentially expressed genes in the control group and ten in the PNS group, seven of which were located on the X chromosome. The ubiquitin-specific peptidase 9 X-linked gene, important for brain development, was among these. Comparison of males to females across all groups revealed 58 differentially expressed genes, with 45 exhibiting higher expression in females. Many of these differentially expressed genes are associated with pathways related to infectious diseases. These findings align with previous research on adult cattle exposed to similar prenatal stress conditions, where minimal differences in amygdala gene expression were observed. To further investigate the implications of prenatal stress and sex differences in cattle, future studies could examine postnatal amygdala size, connectivity, and functional responses to various stimuli.

## 1. Introduction

The amygdala, located within the brain’s temporal lobe, is part of the limbic system and plays a crucial role in how an animal assesses and responds to environmental stimuli. The amygdala processes sensory information, such as olfactory, auditory, visual, and somatosensory information, that comes from other areas of the brain [1]. After processing the stimuli, the amygdala communicates with the brain and limbic system and ultimately can trigger an animal’s fear, anxiety, and stress response [2]. It also plays a prominent role in emotional learning and behavior such as fear memory formation [3].

Proper gene expression within the amygdala is essential for processing stimuli and eliciting the appropriate response. Aberrant gene expression within the amygdala can result in altered behavior, depression, anxiety, and post-traumatic stress disorder [4,5,6], which can lead to long-term emotional and behavioral challenges. Gene expression within the amygdala can be influenced by numerous factors such as sex and prenatal exposures. Neuropeptide gene expression showed dramatic differences between male and female mice [7]. Similar patterns have been observed in other genes, such as DNA damage-inducible delta and methyl CpG binding protein 2 [8,9]. Mice offspring exposed to prenatal stress demonstrated altered gene expression related to chloride transporters and brain-derived neurotrophic factor [10,11]. Similar patterns were observed in the gene calcium voltage-gated channel subunit alpha 1 C expression in mice exposed to dexamethasone prenatally [12]. Additionally, prenatal stress exposure has been linked to an excitatory/inhibitory imbalance in the amygdala, impacting GABAergic neurotransmission and potentially increasing vulnerability to mood disorders [13]. Prenatal stress has been noted to elicit sex-specific effects on gene expression within the amygdala. Sex-specific alterations in the expression of corticotropin-releasing hormone receptors and glucocorticoid receptors have been identified in the amygdala of mice exposed to prenatal stress [14,15]. The amygdala also plays a role in modulating both neuroendocrine and immune responses to infection and inflammation [16,17]. Activation of the amygdala can influence regional inflammatory responses in the brain, highlighting its critical involvement in neuroimmune interaction [18].

The amygdala is crucial for perceiving and responding to stressors that cattle may encounter during various stages of production, including transportation, handling, and changes in their environment, as well as shaping overall behavioral responses. Prenatal stress induced small changes in DNA methylation patterns and gene expression patterns within amygdala tissue from 5-year-old cows [19]. However, the impact of prenatal stress on gene expression within the amygdala at a younger age and sex-specific influences on cattle have not been investigated. Littlejohn et al. [20] found that 28-day-old calves born to prenatally stressed dams exhibited more temperamental behavior compared to calves from control dams, suggesting that prenatal stress may influence offspring temperament, possibly through alterations in gene expression within the amygdala. Thus, the objective of this research was to investigate the effect of prenatal transportation stress and sex on gene expression within the amygdala of 25 d old Brahman bull and heifer calves.

## 2. Methods

### 2.1. Animal Handling

All procedures followed the Guide for the Care and Use of Agricultural Animals in Research and Teaching [21] and were approved by the Texas A&M AgriLife Research Agricultural Animal Care and Use Committee (Animal Use Protocol 2017-035A).

Purebred Brahman bull and heifer calves whose dams were either (a) exposed to transportation during gestation as a prenatal stressor or (b) not transported during gestation were used in this study. Details of this stress model were previously described by Lay et al. [22], Price et al. [23], and Littlejohn et al. [20] Semen from a single bull was used to artificially inseminate Brahman cows. The pregnant cows were then separated into two groups randomly with respect to temperament assessment, age, and parity. One group was transported in a trailer for a 2 h duration at 60 ± 5, 80 ± 5, 100 ± 5, 120 ± 5, and 140 ± 5 d of gestation. In brief, during each transportation event, cows were transported in the same 3-section trailer (2.4 by 7.3 m) that was towed by a three-quarter ton truck driven by the same person each time on smooth paved highways for a total of 2 h at an average speed of 75 km/h (150 km total distance). The non-transport group was the control. Transported and control cows were maintained together under the same environmental and nutritional conditions in the same coastal bermudagrass and ryegrass pastures at the Texas A&M AgriLife Research and Extension Center in Overton, TX, USA. The diet for the cows consisted of ad libitum coastal bermudagrass in pastures during the summer and fall. The same pastures were overseeded with rye (*Secale cereale*) and ryegrass (*Lolium multiflorum*). Cows were supplemented with coastal bermudagrass (*Cynodon dactylon*) hay and a 3:1 corn/soybean meal mix as required depending on forage quality and availability. The bull and heifer calves born from the transport group were the prenatally stressed groups (PNS) and calves born to the non-transport group (control). At 25 ± 2 d of age, 8 bull and heifer calves from each group were humanely euthanized via barbiturate overdose, and the right and left amygdalae were dissected from the brain, snap-frozen in liquid nitrogen, and then stored at −80 °C until time for analysis.

### 2.2. RNA Extraction

Amygdala samples (200 mg) from each calf were shipped on dry ice to Zymo Research Corp (Irvine, CA, USA). For extraction of the RNA from the tissue, 1 mL of DNA/RNA Shield from Zymo Research was added to frozen amygdala tissue samples, allowing the samples to thaw while immersed in the protective solution. Once defrosted, the samples were transferred into bead beating tubes containing a 2.0 mm zirconium bead lysis matrix and homogenized using a Precellys Evolution homogenizer (Bertin Technologies, Montigny-le-Bretonneux, France). A 200 μL aliquot of the resulting lysate was then subjected to protein digestion by incubating it with Proteinase K and a dedicated digestion buffer for at least 30 min at room temperature. Following this enzymatic treatment, the samples underwent total RNA purification using the KingFisher Flex automated system from Thermo Fisher Scientific (Waltham, MA, USA), employing reagents from Zymo Research’s Quick-RNA MagBead kit. Subsequent to elution, the purified RNA underwent an additional DNase I treatment and clean-up step using Zymo’s RNA Clean & Concentrator kit (Zymo Research Corporation, Irvine, CA, USA) to remove any residual DNA contaminants and concentrate the RNA.

### 2.3. Library Preparation and Sequencing

Library preparation was performed using the Zymo-Seq RiboFree Total RNA Library Prep Kit Zymo Research Corporation, Irvine, CA, USA). In brief, the isolated RNA was reverse-transcribed into cDNA followed by ribosomal RNA depletion. Partial P7 adapter sequences were ligated to the 3′ end of the cDNA followed by second-strand synthesis and partial P5 adapter ligation to the 5′ end of the DNA. Libraries were then amplified. Successful library construction was confirmed with Agilent’s (Santa Clara, CA, USA) D1000 ScreenTape Assay on TapeStation. RNA-Seq libraries were sequenced on an Illumina (San Diego, CA, USA) NovaSeq to a sequencing depth of at least 30 million read pairs (150 bp paired-end sequencing) per sample. The program MultiQC (v1.25.1) [24] was used to inspect the quality of sequences.

### 2.4. Sequence Alignment and Differential Expression Analysis

Adapter and low-quality sequences were trimmed from raw reads using Trim Galore! v0.6.6 (https://github.com/FelixKrueger/TrimGalore, accessed on 10 March 2023). Trimmed reads were aligned to the most recent *Bos taurus* genome (ARS-UCD1.3) [25] utilizing the Spliced Transcripts Alignment to a Reference (STAR) computer program [26]. Binary alignment map files for each sample were produced by STAR and were subsequently processed with the program HT-Seq [27] to generate gene counts. Gene counts were then compiled in a comma-delimited file for statistical analysis.

To assess the presence of outliers in the RNA-seq data, multiple methods were employed. Initially, a visual inspection of the MDS plot was conducted to identify potential outliers. Following this, two robust algorithms were applied: the PCAGrid and PCAHubert functions. These methods, as detailed by Chen et al. [28], provide objective means of detecting outliers in high-dimensional RNA-seq data. In brief, these robust PCA methods (PCAGrid and PCAHubert) calculate distances to identify outliers. Both use orthogonal distance (OD) and score distance (SD) to measure sample deviation from the PCA subspace and distance from the center within the subspace, respectively. PCAGrid uses a projection pursuit technique, while PCAHubert combines this with a robust covariance estimation method. For both methods, cutoff values are typically determined based on the 97.5% quantile of the chi-square distribution. Samples exceeding these cutoffs are classified as outliers.

Further statistical analyses for differential expression were conducted using the R package edgeR (v4.0). Raw read counts were imported into R, followed by filtering out genes with zero counts across all samples. The remaining gene counts were then normalized using the trimmed mean of M-values [29] method to account for differences in library sizes and RNA composition between samples. Tagwise dispersion was estimated using the empirical Bayes method, which borrows information across genes to improve accuracy. A negative binomial generalized linear model was fitted to the normalized count data for each gene, with the design matrix including the factors of sex and treatment. Differential expression testing was performed using a likelihood ratio test for each gene, comparing the full model to a reduced model without the contrast of interest. *p*-values were adjusted for multiple testing using the Benjamini–Hochberg [30] method to control the false discovery rate. Genes were considered differentially expressed if they met the criteria of an adjusted *p*-value of 0.20. Log fold changes were calculated using edgeR’s empirical Bayes estimation procedure, which employs a negative binomial model for count data. The log fold changes represent the difference in log2-transformed counts per million (CPM) between groups, normalized for library size. edgeR applies empirical Bayes shrinkage to moderate these estimates, stabilizing log fold changes for genes with low counts or high variability. This approach balances observed changes with an overall mean log fold change, resulting in more robust estimates.

### 2.5. Gene Enrichment Analysis

Differentially expressed genes from each comparison were input into KOBAS-i [31] for gene enrichment analysis. KOBAS-i employs the hypergeometric test and Fisher’s exact test to evaluate the significance of pathway enrichment for differentially expressed genes. Pathways were considered significant with an FDR ≤ 0.05.

## 3. Results and Discussion

### 3.1. Sequencing and Mapping Quality

The average number of sequences produced across the 32 samples was 53.8 million. The average percentage of reads that were uniquely mapped during the alignment process across the remaining samples was 94.24%.

### 3.2. Outlier Analysis

**Figure 1:**
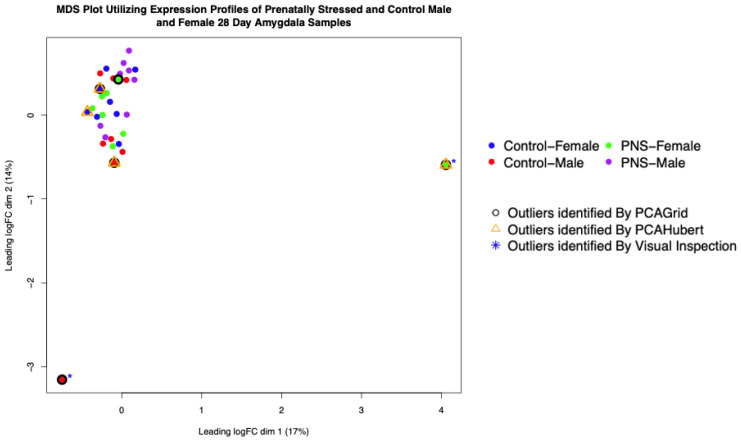
Multidimensional scaling plot of distances between gene expression profiles from amygdala tissue of 25-day-old male and female prenatally stressed (PNS) and control Brahman calves.

After filtering for genes with no gene expression across all samples, 24,574 genes remained for further testing. A multidimensional scaling plot (MDS) was constructed utilizing the normalized gene counts of the 32 samples (Figure 1). There was a lack of clustering between the groups (treatment or sex) and the dispersion estimate was 0.07. In the PCA plot (Figure 1), two samples stood out visually as outliers: a control male and a PNS female. The control male had significantly fewer reads produced (36.5 million), but the PNS female showed no major differences in mapping quality or statistics. The rPCA analysis resulted in the designation of those samples and three additional samples (PNS female and control male and control female) as outliers. The cutoff values for outlier detection were 1.414095 × 10^−8^ for orthogonal distance and 7.034 for scored distance. These samples fell outside the calculated values (Supplementary Table S1), indicating they were significantly different from other samples in their treatment groups in terms of overall gene expression profiles [28]. The PCAHubert approach identified the control male with reduced reads and then three additional samples (two control females and one control male). The cutoff values for outlier detection were 9074.45 for orthogonal distance and 2.241 for scored distance. These samples fell outside the calculated values (Supplementary Table S1). Figure 1 presents an MDS plot with the identified samples from all methods.

To evaluate the impact of outliers on gene expression analysis, preliminary analyses were conducted with and without the inclusion of identified outlier samples. The number of DEGs identified in each comparison is summarized in Table 1. Notably, the removal of outliers, regardless of the method used, resulted in a significant reduction in the number of DEGs. This substantial change in DEG counts indicates that the outlier samples had a disproportionate influence on the comparisons, potentially skewing the results and obscuring the true effects of treatment or sex in the analyses.

**Figure 2:**
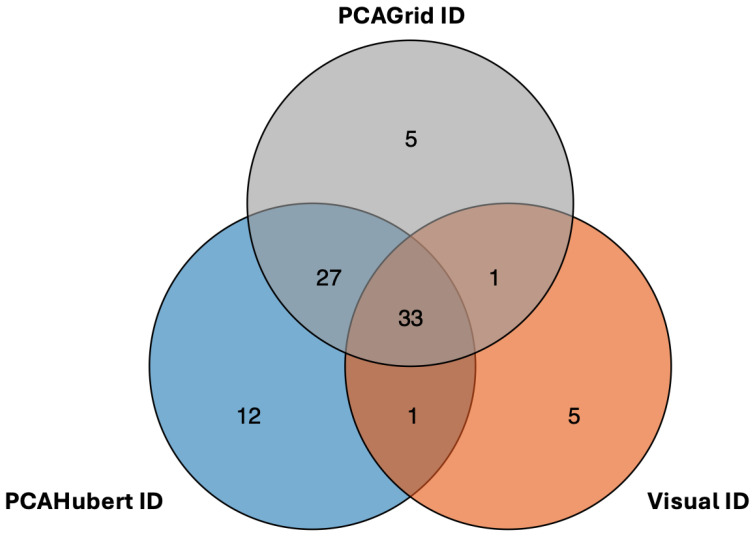
Overlap and comparison of differentially expressed genes in male vs. female calves across various outlier removal methods.

Figure 2 features the comparison of DEGs identified through various outlier removal methods, specifically focusing on the comparison between males and females, which yielded the highest number of DEGs. Each circle in the diagram corresponds to a distinct outlier detection approach, highlighting both the shared and unique DEGs identified by each method. The overlap among the circles indicates 33 DEGs that were consistently identified across multiple methods, suggesting that these genes are robustly differentially expressed regardless of the outlier removal strategy employed. In contrast, the unique DEGs identified by each method highlight the method-specific sensitivities and biases, such as the ability of rPCA and PCAHubert to detect genes influenced by subtle variations that visual inspection might overlook.

The PCAGrid method was chosen for outlier removal due to its robust statistical approach and comprehensive detection capabilities. It effectively identified samples that deviated significantly from others in their treatment groups, based on statistically determined cutoff values on the outlier map. In contrast, PCAHubert failed to identify one sample that was clearly a visual outlier on the MDS plot, which may indicate limitations in sensitivity. PCAGrid demonstrated superior sensitivity in detecting samples that could disproportionately influence the results. Removing these outliers resulted in a significant reduction in the number of DEGs, indicating that PCAGrid helps reveal true biological effects by minimizing noise. The consistent identification of 33 DEGs across multiple methods underscores their robustness as true detections, validating PCAGrid’s ability to capture genuine biological differences. While methods like rPCA and PCAHubert have specific sensitivities, PCAGrid was selected to balance between sensitivity and specificity. Additionally, PCAGrid successfully identified both outlier samples that were previously detected through visual inspection. Consequently, the five samples identified by the PCAGrid algorithm were excluded from the subsequent comparisons.

### 3.3. Effect of Prenatal Stress Within Sex

The treatment comparison for both males and females yielded no significant DEGs (FDR ≤ 0.20). Due to the limited number of differentially expressed genes identified, a pathway analysis could not be performed for these comparisons. It is possible that while transportation is stressful enough to elicit a physiological stress response in the dams [23], it might not be stressful enough to cause gene expression within the amygdala of the offspring. When expression differences have been observed in other work, various stressors were imposed for extended periods of time. Boersma et al. [10] exposed rats to different stressors, including extended periods of restraint, swim tests, cold exposure, and social stress. Unpredictable shock stress resulted in gene expression differences within the amygdala of mice [32]. Prenatal stress induced sex-specific effects in mice whose dams were exposed to variable stressors, including periods of restraint, forced swimming, and elevated platform exposure [33]. The timing of stress during gestation may affect the outcome of prenatal stress in the offspring [34]. Stress at a later point in gestation might cause region-specific effects on the developing brain [35]. Stressors applied later during gestation might have a more pronounced effect on gene expression within the amygdala. However, stress later in gestation may lead to increased pregnancy loss.

### 3.4. Effect of Sex Within Treatment

Within treatment groups, the sex comparison resulted in few DEGs (FDR ≤ 0.20). Within the control group, seven genes were differentially expressed (Table 2). There were 10 DEGs between the PNS males and females (Table 3). Of those, seven were located on the X chromosome. Substrates from ubiquitin specific peptidase 9 X-linked (*USP9X,* FDR = 8.68 × 10^−4^) are involved in neurodevelopmental signaling pathways. *USP9X* removes ubiquitin molecules from its substrate proteins, thereby protecting them from degradation and stabilizing their levels. The protein product of USP9X plays crucial roles in brain development and function, including neuronal migration, axon growth, and synaptic development [36]. Unlike other X-linked genes, *USP9X* escapes X inactivation, meaning that both copies are expressed in females [37]. Increased *USP9X* expression in females, due to its escape from X-inactivation, has been observed previously. In mice, higher expression of *USP9X* protein was observed in brain tissue from females [38]. This dual expression is crucial for normal development, as even partial loss of *USP9X* expression or function in females can lead to neurodevelopmental disorders [39].

Eukaryotic translation initiation factor 2 subunit gamma (*EIF2S3*) had lower expression in males in both the PNS and control comparisons, with log fold changes of −0.494 and −0.466, respectively (Table 2 and Table 3). Eukaryotic translation initiation factor 2 subunit gamma is a part of the eIF2 complex that helps initiate protein synthesis. Similarly to *USP9X*, *EIF2S3* has been observed to escape X-inactivation in both mice and humans, potentially leading to higher expression levels in females compared to males [40,41]. Although the amygdala was not investigated, females exhibited significantly higher levels of *EIF2S3* mRNA expression than males in the hippocampus and paraventricular nucleus of mice [42].

Additionally, the shroom family member 2 (*SHROOM2*) had increased expression in males in both the PNS and control groups (logFC = 0.911 and 0.699, respectively). The product of *SHROOM2* is involved in endothelial morphogenesis [43]. Elevated levels of *SHROOM2* expression have been observed in the cardiac muscle of male mice and male *H. sapiens* relative to females [44]. While *SHROOM2* itself has not been identified to have a specific role in the brain, other members of the *SHROOM* gene family have been implicated in neurulation [45]. Unlike *USP9X* and *EIF2S3*, which have been observed to escape X-inactivation, it is currently unknown whether *SHROOM2* escapes X-inactivation.

### 3.5. Across Group Comparison

Comparisons were also made across treatment and sex. When comparing control to PNS, disregarding sex, there was a single gene that was differentially expressed (FDR = 0.059): cofilin 2 (*CFL2*). Cofilin proteins can regulate synaptic function [46]. Exposure to stressful environments disrupts actin dynamics through cofilin inactivation, leading to altered synaptic function and anxiety-related behaviors; a study found that neonatal social isolation in juvenile rats inactivated cofilin [47]. While stress can induce the formation of cofilin–actin stress rods within cells [48], the effect of prenatal stress on *CFL2* expression is not known.

**Figure 3:**
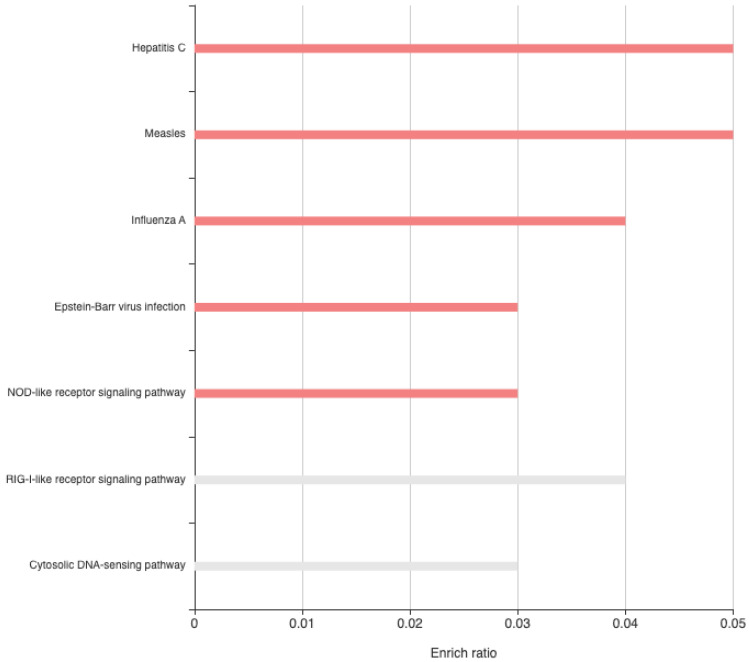
Gene enrichment analysis of differentially expressed genes in amygdala tissue between males and females.

When disregarding treatment, 58 genes were differentially expressed between males and females (Supplementary Table S2). The majority *(n* = 45) showed increased expression in females compared to males. The LIM homeobox 1 gene had increased expression in males relative to females, a pattern that was observed within the pig amygdala [49]. General differences in gene expression within the amygdala between males and females have been observed in genes related to the circadian clock and energy metabolism in mice and in humans [50]. However, gene enrichment analysis revealed that many of the differently expressed genes are involved in infectious disease (viral) KEGG pathways, including Hepatitis C and Influenza A (Supplementary Table S3, Figure 3). The central amygdala modulates neuroendocrine, febrile, and behavioral responses to HSV-1 infection, as infected rats showed reduced HPA axis activation, fever, motor hyperactivity, and aggressive behavior [17]. Additionally, the amygdala demonstrates neuronal activity changes, measured by intracerebral electroencephalography and c-fos abundance, in response to lipopolysaccharide challenges [51]. Most studies characterizing the immune response in cattle focus on blood samples. Consequently, the potential role of the amygdala in beef cattle immune response remains unknown. However, sex differences in immune responses have been observed. Notably, beef cattle exhibited sex differences in response to viral, bacterial, and lipopolysaccharide challenges [52,53]. The current project identified sexually dimorphic expression of amygdala genes in calves at 25 days of age. Sexually dimorphic responses of older beef cattle to stress have been reported. Heifers and bulls at 8–9 months of age differed in response to provocative systemic lipopolysaccharide challenge [53]. Specifically, the LPS-induced increase in heart rate was greater in heifers than bulls whereas sickness scores were less in heifers than bull calves. In addition, heifers exhibited a greater febrile response, an enhanced tumor necrosis alpha (TNF-α) response, and neutrophilia, while bulls had more leukocytes and a greater IFN-γ response. Sexual dimorphic responses to prenatal stress have been reported in goats and pigs [54,55]. For example, 10-week-old female but not male pigs whose mothers were exposed to a social stressor during gestation had increased anxiety-like behavior as adolescents and as mothers [55].

While gene expression offers valuable insights, investigating additional factors might further explain the interplay between prenatal stress, sex, and amygdala function in cattle. Prenatal stress has resulted in size differences in the amygdala [56], altered connectivity [57,58], and reduced amygdala neuron excitability, as well as altered socioemotional behavior in *H. sapiens* [59]. The current project identified sexually dimorphic expression of amygdala genes in calves at 25 days of age. This age was selected to obtain the amygdala for analysis because a prior project determined that the temperament of beef calves could be assessed at 28 days of age and that a divergence in temperament was already detectable at 28 days of age in prenatally stressed calves [20]. Specifically, prenatally stressed calves were more temperamental than control calves through weaning at 6 months of age. At 8 months of age, prenatally stressed and control beef calves differed in innate immune response. Specifically, prenatally stressed calves had elevated resting pro-inflammatory cytokine concentrations and a greater interferon gamma (IFN-γ) response to an endotoxin challenge [60]. Sex differences have also been observed in prenatally stressed males and females for measurements such as amygdala degree centrality and amygdala volume [61,62]. Sex differences may be further highlighted by monitoring amygdala activity in response to various stimuli, including immune system activation [63].

Gene expression patterns were analyzed in 5-year-old cattle that had been exposed to the same treatment conditions as the calves in the current study. Baker et al. [17] reported minimal differences between PNS and control cows in amygdala tissues. It was hypothesized that any differences potentially present at a younger age during critical developmental periods may have diminished over time. This led to a shift in focus towards examining expression patterns at an earlier stage of development. However, similar results were observed even when investigating calves at just 25 days of age. The study revealed very minimal differences in gene expression patterns within the amygdala, mirroring the findings from the adult cattle. The lack of differences could be attributed to various factors, including the specific timing of stress exposure during gestation and the severity of the stressors involved. While gene expression offers valuable insights, investigating additional factors might further explain the interplay between prenatal stress, sex, and amygdala function in cattle. Although the cows in this study were managed under the same environmental conditions, future research could benefit from explicitly measuring and accounting for potential variations in maternal health, nutrition, and individual stress responses. These factors could influence fetal development and potentially modulate the effects of prenatal stress on amygdala function.

## 4. Conclusions

This study is a continuation of ongoing research into the impact of prenatal stress on gene expression patterns within the amygdala and other tissues of tropically adapted beef cattle. The findings from this research have important implications for animal welfare and management practices in cattle production, as understanding the molecular consequences of prenatal stress can inform strategies to mitigate its negative effects on cattle health, behavior, and productivity. Successful mitigation of stressors associated with postnatal issues, such as respiratory disease in feedlot cattle, depends upon the identification of early-life factors that affect lifetime health, well-being, and performance [64]. An initial challenge with this dataset was the proper identification and management of outliers in gene expression data. The effects of outliers and various outlier identification methods were systematically evaluated, ultimately leading to the selection of a method that balanced sensitivity with specificity. The influence of prenatal stress on the amygdala in both male and female Brahman calves appears to be negligible. Although limited, some differences between sexes were observed, particularly involving genes on the X chromosome. Further exploration into amygdala size, connectivity, and responses to stimuli presents a challenging but alternative approach for more comprehensive understanding of how prenatal stressors and sex-specific factors influence amygdala function in cattle.

## Figures and Tables

**Table 1:** Number of differentially expressed genes (FDR ≤ 0.20) for each comparison before and after removal of outliers.

Comparison	All Samples	Visual Outlier Removal	PCAGrid Outlier Removal	PCAHubert Outlier Removal
PNS ^1^ vs. control females	134	0	0	0
PNS vs. control males	2	1	0	0
Males vs. females control	8	7	7	12
Males vs. females PNS	138	9	10	10
Males vs. females	48	40	58	73
PNS vs. control	6	0	2	2

^1^ Prenatally stressed.

**Table 2:** Genes that were differentially expressed within amygdala tissue of males vs. females of the control group at 25 days of age.

Gene Name	Chromosome	LogFC ^1^	FDR
shroom family member 2 *	X	0.699	1.16 × 10^−4^
FKBP prolyl isomerase family member 6	25	3.365	1.92 × 10^−4^
mitotic arrest deficient 2-like 1	NKLS02000723.1	−1.456	5.08 × 10^−4^
eukaryotic translation initiation factor 2 subunit gamma *	X	−0.466	0.016
interferon-induced protein 44-like	3	−2.403	0.147
interferon-induced protein with tetratricopeptide repeats 1	26	−3.373	0.194
MX dynamin like GTPase 2	1	−2.874	0.194

^1^ LogFC = Logarithmic Fold Change: positive (negative) fold change indicates that males had increased (decreased) expression relative to females. * Identifies genes exhibiting differential expression in both the male vs. female comparisons within the control group and the male vs. female comparisons within the prenatal stress group.

**Table 3:** Genes that were differentially expressed within amygdala tissue of males vs. females of the prenatally stressed group at 25 days of age.

Gene Name	Chromosome	LogFC ^1^	FDR
shroom family member 2 *	X	0.911	2.43 × 10^−10^
eukaryotic translation initiation factor 2 subunit gamma *	X	−0.494	6.17 × 10^−4^
ubiquitin specific peptidase 9 x-linked	X	−0.360	8.68 × 10^−4^
ATPase sarcoplasmic/endoplasmic reticulum Ca^2+^ transporting 3	19	−0.794	0.021
glypican 3	X	1.35	0.085
fez family zinc finger 2	22	−0.662	0.093
ENSBTAG00000051077	X	−4.562	0.093
arylsulfatase l	X	0.967	0.152
matrix remodeling-associated 5	X	0.893	0.152
mitotic arrest deficient 2-like 1	NKLS02000723.1	−1.01	0.168

^1^ LogFC = Logarithmic Fold Change: positive (negative) fold change indicates that males had increased (decreased) expression relative to females. * Identifies genes exhibiting differential expression in both the male vs. female comparisons within the control group and the male vs. female comparisons within the prenatal stress group.

## Data Availability

The raw data supporting the conclusions of this article will be made available by the authors on request.

## Supplementary Materials

The following supporting information can be downloaded at: https://www.mdpi.com/article/10.3390/biology13110915/s1, Table S1: Orthogonal distances and scored distances for samples from the PCAGrid and PCAHubert methodologies; Table S2: Differentially expressed genes between males and females disregarding treatment; Table S3: Significant KEGG pathways identified via KOBAS analysis of differentially expressed genes between males and females.
